# Lipid Metabolism in Cancer: The Role of Acylglycerolphosphate Acyltransferases (AGPATs)

**DOI:** 10.3390/cancers14010228

**Published:** 2022-01-04

**Authors:** Angeliki Karagiota, Georgia Chachami, Efrosyni Paraskeva

**Affiliations:** 1 Laboratory of Biochemistry, Faculty of Medicine, University of Thessaly, BIOPOLIS, 41500 Larissa, Greece; akaragi@uth.gr (A.K.); ghah@uth.gr (G.C.); 2 Laboratory of Physiology, Faculty of Medicine, University of Thessaly, BIOPOLIS, 41500 Larissa, Greece

**Keywords:** lipids, metabolism, AGPAT, LPAAT, phosphatidic acid, cancer

## Abstract

**Simple Summary:**

Rapidly proliferating cancer cells reprogram lipid metabolism to keep the balance between fatty acid uptake, synthesis, consumption, and storage as triacylglycerides (TAG). Acylglycerolphosphate acyltransferases (AGPATs)/lysophosphatidic acid acyltransferases (LPAATs) are a family of enzymes that catalyze the synthesis of phosphatidic acid (PA), an intermediate in TAG synthesis, a signaling molecule, and a precursor of phospholipids. Importantly, the expression of AGPATs has been linked to diverse physiological and pathological phenotypes, including cancer. In this review, we present an overview of lipid metabolism reprogramming in cancer cells and give insight into the expression of AGPAT isoforms as well as their association with cancers, parameters of tumor biology, patient classification, and prognosis.

**Abstract:**

Altered lipid metabolism is an emerging hallmark of aggressive tumors, as rapidly proliferating cancer cells reprogram fatty acid (FA) uptake, synthesis, storage, and usage to meet their increased energy demands. Central to these adaptive changes, is the conversion of excess FA to neutral triacylglycerides (TAG) and their storage in lipid droplets (LDs). Acylglycerolphosphate acyltransferases (AGPATs), also known as lysophosphatidic acid acyltransferases (LPAATs), are a family of five enzymes that catalyze the conversion of lysophosphatidic acid (LPA) to phosphatidic acid (PA), the second step of the TAG biosynthesis pathway. PA, apart from its role as an intermediate in TAG synthesis, is also a precursor of glycerophospholipids and a cell signaling molecule. Although the different AGPAT isoforms catalyze the same reaction, they appear to have unique non-overlapping roles possibly determined by their distinct tissue expression and substrate specificity. This is best exemplified by the role of AGPAT2 in the development of type 1 congenital generalized lipodystrophy (CGL) and is also manifested by recent studies highlighting the involvement of AGPATs in the physiology and pathology of various tissues and organs. Importantly, AGPAT isoform expression has been shown to enhance proliferation and chemoresistance of cancer cells and correlates with increased risk of tumor development or aggressive phenotypes of several types of tumors.

## 1. The Role of Lipids in Normal and Cancer Cell Physiology

### 1.1. Overview of Lipid Metabolism

Lipids are a heterogeneous group of water-insoluble molecules, which includes Fatty Acids (FA), acylglycerols (e.g., tri-, di-, and mono-acylglycerides: TAG, DAG, MAG), phosphoglycerides (PG), sterols, and sphingolipids. Lipids possess several functions at cellular and organismal levels, making them critical for survival. Cellular FAs originate from exogenous uptake or de novo synthesis and are mainly used as substrates for FA oxidation and energy production or synthesis of TAGs and energy storage [1]. PGs, along with sterols and sphingolipids, are the main structural elements of biological membranes. Lipids also take part in signaling, operating as second messengers and hormones [2].

**Figure 1:**
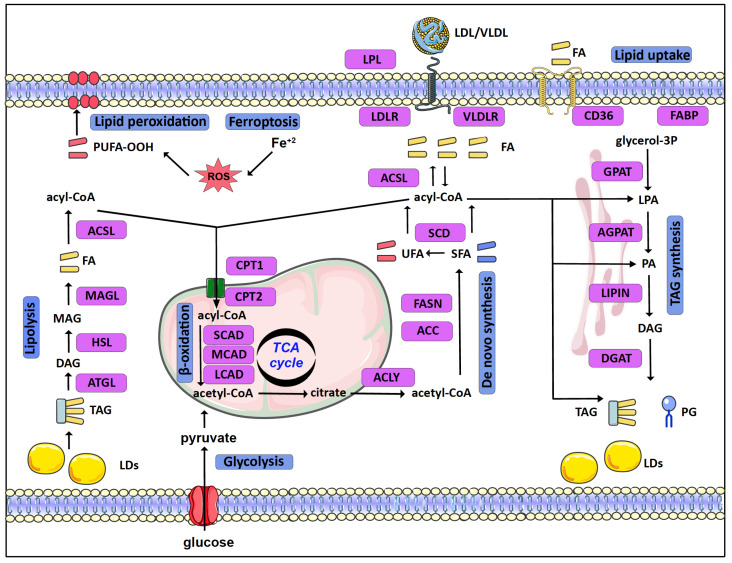
Schematic overview of lipid metabolism in normal as well as cancer cells. Lipid uptake of free fatty acids (FA: saturated, unsaturated, and/or polyunsaturated fatty acids) or Low-/Very-Low-Density Lipoproteins (LDL/VLDL) is facilitated by lipoprotein lipase (LPL), receptors (LDLR/VLDLR, CD36), and FA binding proteins (FABP). De novo synthesis, fueled by acetyl-CoA derived from citrate produced in the TCA cycle, creates saturated FA (SFA) and unsaturated FA (UFA). FAs in the form of acyl-CoA are used for the synthesis of triacylglycerides (TAG) and phosphoglycerides (PG) and stored in lipid droplets (LDs). LDs can be broken down to FAs by lipolysis. FAs, after conversion to acyl-CoA, can also be transported to mitochondria and broken down to acetyl-CoA by β-oxidation. The production and oxidation of polyunsaturated FA (PUFA) in the presence of Fe^2+^ and oxidants (ROS) is directly related to cell death by ferroptosis. Lipid metabolism-related enzymes are shown as purple rectangles (see text for further details). (Abbreviations: PA (Phosphatidic Acid), Lysophosphatidic Acid (LPA), Diacylglycerol (DAG), Monoacylglycerol (MAG), short-chain acyl-coenzyme A dehydrogenase (SCAD), medium-chain acyl-coenzyme A dehydrogenase (MCAD), and long-chain acyl-coenzyme A dehydrogenase (LCAD)).

Mammalian cells acquire lipids, provided by dietary sources, the liver, or adipocytes, directly from the bloodstream as free FA or as complexes in Low- or Very-Low-Density Lipoproteins (LDL or VLDL, respectively). Lipid uptake is facilitated by specific receptors or binding proteins, such as FABPs, CD36, VLDL, and LDL receptors [3]. De novo synthesis is also an important source of lipids, especially in specific tissues such as the adipose tissue and the liver [4]. Citrate, produced in the tricarboxylic acid (TCA) cycle, is the main source of acetyl groups for FA biosynthesis. Cytoplasmic acetyl-CoA and malonyl-CoA are used to synthesize palmitate, which can be further processed (e.g., elongated, desaturated) by specific enzymes mainly in the endoplasmic reticulum (ER) (Figure 1).

In eukaryotes, TAGs are mainly synthesized in the glycerol phosphate (Kennedy) pathway, in four consecutive reactions, through a stepwise addition of fatty acyl groups [5]. The pathway starts as glycerol-3-phosphate acyltransferases (GPATs) convert glycerol-3-phosphate (G3P) to lysophosphatidic acid (LPA) or monoacylglycerol-3-phosphate. In the second reaction, LPA is transformed to phosphatidic acid (PA) or diacylglycerol-3-phosphate by AGPATs. Subsequently, a family of proteins with Mg^2+^-dependent phosphatidic phosphatase activity, called phosphatidic acid phosphohydrolases (PAPs) or lipins (LPINs), catalyzes the dephosphorylation of PA and the formation of diacylglycerol (DAG) [6]. Thereafter, DAG is acylated by diglyceride acyltransferases (DGATs) in order to form TAG [7].

TAGs are stored in lipid droplets (LDs). LDs consist of a hydrophobic core of TAGs and sterols surrounded by a more hydrophilic phospholipid layer. LDs vary in size and number and their biogenesis and function are tightly controlled. Stored TAGs can be broken down to free FA by lipolysis. Adipose triglyceride lipase (ATGL) initiates degradation of TAG to DAG. Hormone-sensitive lipase (HSL) further hydrolyzes DAG to MAG, while MAG lipase (MAGL) hydrolyzes MAG to free FA and glycerol [5,8,9]. Finally, activated FAs are connected to carnitine by CPT1 (carnitine palmitoyl-transferase 1), transported to mitochondria, and broken down by β-oxidation [10].

### 1.2. Reprogramming of Lipid Metabolism in Cancer

Rapidly proliferating cancer cells reprogram their metabolism in order to meet increased energy and macromolecule requirements. To this end, lipidomic remodeling is a common feature of carcinogenesis (recently reviewed in [1,3,11]) (Figure 1).

To begin with, cancer cells upregulate de novo synthesis of FA [12,13]. ATP-citrate lyase (ACLY) and acetyl-CoA carboxylase (ACC), the enzymes that convert citrate to acetyl-CoA and subsequently to malonyl-CoA in the cytoplasm, are upregulated in a number of cancers such as lung, liver, ovarian, and colorectal cancer. Likewise, FASN (fatty acid synthase) shows increased expression in cancers such as breast and prostate cancer, and a high activity of FASN has been correlated with poor disease prognosis [14,15,16]. These changes are mediated via the activation of lipid metabolism gene-related transcription factors, such as SREBP [17,18,19]. Importantly, in contrast to FA uptake, de novo synthesis of FA provides cancer cells with the flexibility to control their lipid content and composition, via the regulation of specific enzymes responsible for the synthesis of saturated as well as unsaturated FAs. For example, stearoyl-CoA desaturase (SCD), an enzyme responsible for the synthesis of monounsaturated FAs, has been found elevated in cancer cells [13,20].

In addition to de novo synthesis, upregulation of lipid uptake by VLDL receptors and lipoprotein lipase (LPL) in breast cancer cells contributes to increased lipid uptake [21]. The increased FA uptake and synthesis in cancer cells can lead to an excess of free FAs and lipotoxicity. This risk is eluded by converting FA to TAGs and storing them in LDs, serving the double purpose of shielding the cell from lipotoxicity and providing fuel for energy production [9,22,23]. Several enzymes of the TAG synthesis and LD formation pathways are enhanced in tumors. GPAT2, a mitochondrial GPAT isoform primarily expressed in testes, was linked with breast cancer proliferation and migration [24], while AGPATs are also deregulated in a variety of cancers, as discussed in detail below. Interestingly, the accumulation of LDs is further increased in the hypoxic tumor microenvironment via the Hypoxia Inducible Factors (HIFs) [25], as part of the adaptation of cancer cells to the challenging tumor microenvironment. Accordingly, HIF-1 mediates the induction of genes involved in TAG synthesis [26,27] and LD formation [28,29,30]. HIF-1 further promotes lipid storage via the repression of FA oxidation [31,32].

Despite the preference of cancer cells towards lipid accumulation, the lipids stored in LDs can serve as fuels to sustain cancer cell proliferation and support tumor metastasis. To this end, upregulation of lipolysis enzymes is associated with aggressive tumor behavior [11], while β-oxidation is a significant energy source that is important for survival in many cancers [33,34].

Notably, the readjustment of lipid metabolism in cancer cells not only significantly supports the increased needs of proliferating tumor cells but can also protect them from cell death. Ferroptosis is a form of cell death that results from iron-dependent production of high amounts of reactive oxygen species (ROS) [35,36]. Lipid metabolism is directly related to the mechanism of ferroptosis via multiple ways. Specific enzymes of lipid metabolism, such as acyl-CoA synthetases (ACSL), and lipids, such as polyunsaturated fatty acids (PUFA), play important roles in the generation of lethal lipid peroxides. Highly proliferative cancer cells need higher levels of iron and membrane lipids, which make them more susceptible to ferroptosis [1,37,38]. Cancer cells overcome this challenge, as increased de novo synthesis of FA provides cancer cells with the flexibility to control their PUFA content via the upregulation of synthesis of saturated FAs and by storing PUFA in TAGs [1].

## 2. The AGPAT Family

**Figure 2:**
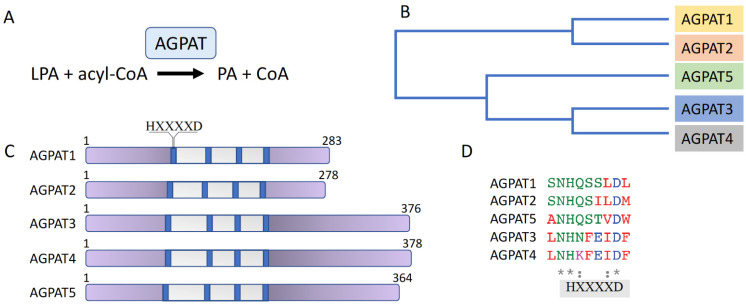
The AGPAT enzyme family. (**A**) AGPATs catalyze the conversion of LPA to PA. (**B**) Phylogenetic tree of AGPAT1-5. (**C**) Schematic representation of AGPAT isoform domain structure. The acyltransferase region is shown in grey; the four acyltransferase motifs are shown in blue. The HXXXXD signature, conferring catalytic activity, is present in the first acyltransferase motif of all isoforms. (**D**) Amino acid sequence alignment of the region containing the AGPAT isoforms’ HXXXXD signature. Conserved amino acids are marked by an asterisk (*).

AGPATs catalyze the synthesis of PA in the second step of the TAG synthesis pathway. They are a family of five membrane-bound acyltransferases (AGPAT1-5) that specifically use acyl-CoA as acyl-donor and lysophospholipid as acyl-acceptor. The five AGPAT family isoforms are encoded by different genes. Amino acid sequence alignment of the human proteins shows that AGPAT1 and AGPAT2 share 75% of amino acid sequence homology and 35% of amino acid identity [39]. Likewise, AGPAT3 and AGPAT4 are evolutionarily closer to each other than to the other isoforms, sharing 75% of homology and 61% of identity [40,41], while the AGPAT5 isoform has the least overall homology with the rest of the family members, being about 38% homologous and 20% identical to AGPAT3 and AGPAT4 [42]. AGPAT isoforms contain four acyltransferase motifs and an HXXXXD signature, conferring catalytic activity, is present in the first acyltransferase motif of all isoforms (reviewed in [39,43]) (Figure 2).

### 2.1. Substrate Specificity, Membrane Localization, and Tissue Expression of the AGPAT Isoforms

Although all AGPATs catalyze the same reaction in the TAG biosynthesis pathway, the individual AGPAT enzymes are not biochemically or functionally redundant [39]. This becomes evident by the different phenotypes and defects of specific isoform mutations or loss of expression, with the best characterized being the association of congenital generalized lipodystrophy (CGL) to mutations of the AGPAT2 gene (discussed below). 

This uniqueness of the AGPAT isoforms probably results from their preference for different acyl-CoA species for the production of specific PA pools. Since PA is the common precursor for phospholipids synthesis, the specificity of the different AGPATs for distinct LPA and acyl-CoA molecules defines the fatty acid composition of downstream synthesized PGs. These phospholipids, in turn, determine the structure and properties of the various biological membranes, have a role in signaling, and participate in cellular functions [39,41]. Studies on the use of acyl-CoA donors have shown that human AGPAT1 and AGPAT2 have broad acyl donor specificity for acyl-CoA [44,45,46]. AGPAT3 and AGPAT4, on the other hand, display a preference for polyunsaturated fatty acyl-CoA [42,47], and AGPAT3 activity has been shown to be important for the synthesis of PUFA-phospholipids in vivo [48].

**Figure 3:**
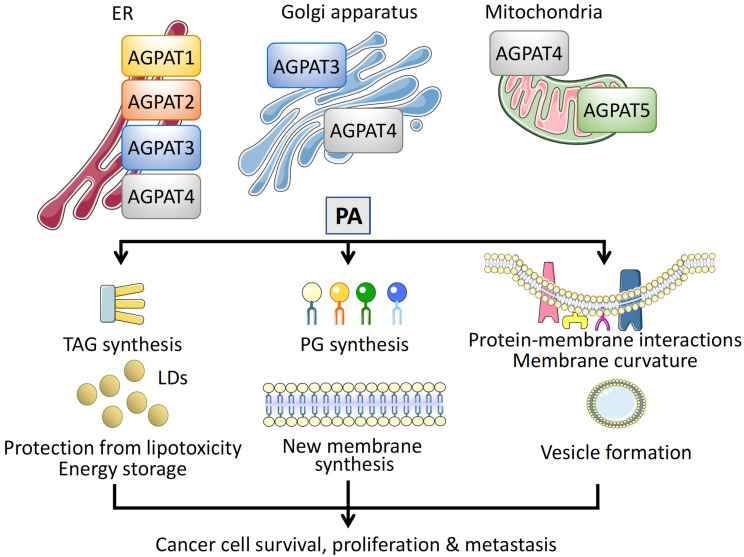
Intracellular localization and function of AGPATs. Different membrane compartments are populated by specific AGPAT isoforms. Isoforms AGPAT1–4 have been localized on the endoplasmic reticulum (ER), AGPAT3, and AGPAT4 on the Golgi apparatus, and AGPAT4 and AGPAT5 on mitochondria. Phosphatidic acid (PA) synthesized via the catalytic activity of AGPATs has multiple roles in cells, promoting cancer cell survival, proliferation, and metastasis. First, PA is an intermediate for the synthesis of TAGs, which are stored in lipid droplets (LDs), protecting cancer cells from free FA toxicity and serving as energy reservoirs. Second, PA is a precursor of phosphoglycerides (PGs), essential for new membrane synthesis in rapidly proliferating cancer cells. Third, insertion of PA regulates protein–membrane interactions, causes membrane curvature, and participates in vesicular transport, mediating cancer cell metastasis.

In addition to the different substrate specificities, the distinct physiological roles of AGPATs are also connected to their subcellular localization [39]. AGPAT1 and AGPAT2 reside exclusively in the ER [44]. AGPAT3 and AGPAT4 have a broader distribution, as AGPAT3 has been localized to the ER and Golgi membranes [42,49,50] and AGPAT4 to the ER, Golgi, and mitochondria [47,50,51]. The fifth family member, AGPAT5, has only been detected on mitochondria [42,50] (Figure 3).

Tissue distribution patterns [52] and mouse model studies [53] reveal a ubiquitous expression of AGPAT1 and AGPAT3. In contrast, AGPAT2, the best studied family isoform, is expressed specifically in adipocytes, liver, and pancreas [44,54]. AGPAT4 is most highly expressed in brain [47,51,55,56] and muscle [57]. Lastly, the highest expression for human AGPAT5 has been found in the testes [42].

### 2.2. Regulation of AGPAT Expression

The multiple and distinct roles of AGPATs in physiology and disease suggest that their expression is regulated under a variety of conditions and, probably, by many transcription factors. However, relatively few studies so far have addressed this issue.

Nutrient availability influences the expression of AGPAT isoforms not only in the liver, the main TAG synthesis site, but also in other organs. FA supplementation increased AGPAT3 mRNA levels during muscle satellite cell differentiation [58]. Accordingly, in mice, acute fasting, which increases the rate of adipocyte TAG hydrolysis and FA release into the bloodstream, led to the increase of mRNA levels of AGPAT2, 3, 4, and 5 in the liver, AGPAT*2* and 3 in the heart, and AGPAT1, 2, and 3 in the brain [59]. Models of tissue injury showed that the epidermis’ experimental barrier disruption caused a tissue-specific induction of AGPAT1, 2, *3*, and 5 mRNA levels [60], although the transcription factors involved in both cases are not known. Additionally, in a model of thoracic irradiation, the expression of AGPAT2 mRNA was increased in the lung of irradiated animals compared to controls [61]. Moreover, hormones most likely participate in the developmental and tissue-specific regulation of AGPAT expression. The expression of AGPAT3 was induced upon treatment of a testicular cell line with β-estradiol [62].

Members of the PPAR family that regulate genes involved in lipid metabolism were among the first factors shown to regulate the expression of AGPATs. An early study reported that treatment with a PPARα agonist increased cardiac AGPAT activity and AGPAT3 mRNA levels of wild-type but not PPARα null mice [53]. The regulation of AGPAT*3* expression by PPARα was further confirmed in mice with heart-specific overexpression of PPARα that had increased cardiac AGPAT3 mRNA levels, along with increased mRNAs of other enzymes involved in TAG synthesis, compared to non-transgenic animals [63]. Furthermore, AGPAT3 mRNA expression was upregulated in differentiating muscle satellite cell culture by agonists of PPARδ. In the same setting, AGPAT3 induction was also achieved by agonists of the AMPK pathway and was further enhanced by the simultaneous treatment with activators of both pathways [58].

HIF-1, in addition to its other target genes, which mediate the adaptation of lipid metabolism under hypoxia [25], has been found to also regulate the transcription of AGPAT2 gene by binding directly to the AGPAT2 promoter in human hepatocellular carcinoma and adenocarcinoma cells. AGPAT2 induction and subsequent increase of AGPAT2 protein levels were abolished upon HIF-1α silencing [27].

GATA-3, a transcription factor required to establish the epidermal barrier, transcriptionally regulates AGPAT5. GATA-3 binds to sites in the first intron of the AGPAT5 gene that are conserved between humans, mice, and rats; mutant GATA-3 mice had decreased expression of AGPAT5 in skin, during epidermal development [64].

Finally, the expression of AGPAT isoforms has been shown to be under the control of miRNAs in cancer cell lines. AGPAT1 is a predicted target of miR-122, a liver-specific miRNA, essential for the maintenance of liver homeostasis and a sensitive biomarker for liver cancer [65]. The miR-340-5p, which is downregulated in cisplatin-resistant osteosarcoma cell lines, targets the 3′ UTR of AGPAT2 and, although it does not affect mRNA levels, decreases the levels of AGPAT2 protein [66]. Accordingly, lncRNA OPI5-AS1, a competing endogenous RNA for miR-340-5p, had the opposite effect and increased AGPAT2 protein levels in osteosarcoma cell lines [67]. Finally, miR-26, a miRNA that is downregulated by estrogen in breast cancer cell lines, was predicted and shown by RT-PCR to target the 3′UTR of AGPAT5 [68].

## 3. The Role of AGPATs in Health and Disease

AGPAT2, the best studied member of the family, is indispensable for adipose tissue development and has been directly associated with type 1 congenital generalized lipodystrophy (CGL). Type 1 CGL, caused by mutations in AGPAT2, is, together with type 2 CGL, resulting from mutations in *BSCL2* gene-encoding seipin, the most common subtype of a heterogeneous autosomal recessive disorder characterized by a generalized lack of body fat, with the exception of the adipose tissue found in skeletal muscle. In addition, CGL patients develop metabolic complications including hyperinsulinemia, insulin resistance, hypertriglyceridemia, diabetes, and hepatic steatosis (reviewed in [54,69]).

The role of AGPAT2 in adipocyte differentiation and lipid biosynthesis has been elucidated by studies in mice, in which knockout of AGPAT2 phenocopied the human CGL phenotype (Cortes et al., 2009), and in vitro studies. AGPAT2^−/−^ mice show excess early mortality (~80%) and develop, like CGL patients, severe adipose tissue abnormalities, insulin resistance, diabetes, and hepatic steatosis [70]. AGPAT2 appears to be essential for adipocyte survival, as newborn AGPAT2^−/−^ mice have normal white and brown adipose tissues, but lack of AGPAT2 causes the death of adipocytes soon after birth [71]. AGPAT2 is also important for lipidome regulation and adipocyte differentiation. AGPAT2 knockout resulted to the unexpected increase of overall PA levels and other phospholipid species via the upregulation of additional PA synthesis pathways [70,71,72]. Indeed, AGPAT2 is necessary for the induction of the adipogenesis regulators C/EBPβ and PPARγ and timely expression of adipocyte-related genes in differentiating adipocytes [72,73]. Accordingly, MEFs from AGPAT2-deficient animals had impaired in vitro adipocyte differentiation [71], while induction of adipogenesis resulted in increased cell death in muscle-derived multipotent cells (MDMCs) from CGL patients or 3T3-L1 preadipocytes lacking AGPAT2 [73]. All of these defects were partially rescued by overexpression of PPARγ or the PPARγ agonist pioglitazone.

In contrast to our extensive knowledge on AGPAT2, the physiological role of the other AGPAT isoforms is less well studied. A mouse model revealed that loss of AGPAT1 is associated with excess embryonic lethality and a high rate of early mortality. Interestingly, despite the homology between AGPAT1 and AGAPT2, AGPAT1^−/−^ mice, although having reduced fat, did not develop lipodystrophy and had low plasma glucose levels [74]. In addition, AGPAT1 knockout caused reproductive defects and affected skeletal muscle development [75]. Importantly, AGPAT1^−/−^ mice exhibited neurological disorders, including seizures [74], while the AGPAT1 gene has been also identified as a gene of interest in studies for neurological diseases such as Parkinson and dementia [76,77].

The physiological role of AGPAT3 appears to be fulfilled through its contribution to the biosynthesis of polyunsaturated phospholipids and regulation of cellular and systemic PUFA levels’ balance. AGPAT3 favors the synthesis of docosahexaenoic acid (DHA)-containing phospholipids and suppresses the activation of SREBP1 and induction of PUFA biosynthetic genes in the liver. DHA-phospholipids synthesized in the liver by AGPAT3 contribute to DHA supply and accumulation in extrahepatic tissues, including the brain, and have an important role in systemic PUFA homeostasis [48]. In a more tissue-specific manner, AGPAT3-mediated synthesis of DHA-containing phospholipids is important in the eyes and the male reproductive system. Accordingly, AGPAT3 knockout mice develop retinal abnormalities and have impaired vision [78], while the induction of AGPAT3 during germ cell development possibly affects sperm cell production [79].

In accordance with the high expression of AGPAT4 in the brain [47], AGPAT4^−/−^ mice have shown significant impairments in spatial learning and memory, possibly due to the reduction in the expression of the NMDA and AMPA glutamic acid receptors, playing a central role in this process [55]. The role of AGPAT4 in higher brain function is supported by a genetic study that identified abnormalities in the AGPAT4 gene locus (a duplication overlapping MAP3K4 and AGPAT4) in patients with non-syndromic intellectual disability [80]. Furthermore, skeletal muscle of AGPAT4^−/−^ mice have altered fiber-type proportion and reduced force generation in specific muscles [57].

The importance of AGPAT3 and AGPAT4 function is extended beyond lipid metabolism to intracellular membrane physiology and vesicular transport. AGPAT3 was shown to regulate COP I-coated vesicle formation and Golgi membrane fission [49,81]. Similarly, the highly homologous AGPAT4 was shown to be a component of a fission-inducing complex. The central protein of this complex, BARS (Brefeldin A ADP-Ribosylated Substrate), is essential for fission in many cell membrane transport processes (reviewed in [41]). AGPAT4 localized on the Golgi is activated by BARS and the conversion of LPA to PA facilitates Golgi membrane fission via a mechanism that involves either the induction of membrane curvature or recruitment of proteins that bind to PA, including BARS itself [50].

The physiological roles of AGPAT5, beyond PA synthesis, remain so far unknown. It is possible that AGPAT5 has an important role in mitochondrial fusion and fission.

## 4. The Role of AGPATs in Cancer

AGPATs influence cancer cell survival and proliferation, as they possess a central part in the biosynthesis of TAGs’ biosynthesis and participate in lipid storage in LDs, which are important for energy storage and prevention of lipotoxicity. In addition, AGPATs, through the synthesis of PA, contribute to the synthesis of phospholipids, which are indispensable for new membrane synthesis in rapidly diving cells as well as the regulation of cell signaling pathways. Finally, recent findings suggest that AGPATs are involved in the regulation of membrane fission and vesicular transport, which is critical for the communication of cancer cells with the tumor microenvironment and cancer metastasis. In addition, an ever-growing list of studies have identified AGPATs as important factors positively or negatively associated with many types of cancers and parameters of tumor biology, including cancer cell proliferation and tumor growth, metastasis, patient classification, and prognosis. Moreover, based on findings showing the differential expression of AGPAT between tumors and normal tissues, combined with results from bioinformatics studies, AGPAT isoforms have been included in prognostic metabolic gene signatures (Table 1).

### 4.1. PA and Cancer Cell Membrane Rearrangements

PA, the product of the AGPAT-catalyzed reaction, is the simplest glycerophospholipid in cells and acts as a precursor for the synthesis of other glycerophospholipids. PA in cells, apart from its de novo synthesis by AGPATs, is also produced by phospholipase D (PLD) and diacylglycerol kinase (DGK). However, it is not clear whether the PA subpopulation generated via each of these pathways serves different purposes within the cell [103].

In cancer cells, PA that is synthesized de novo by AGPATs helps to support the increased demands of membrane biogenesis. In addition, although PLD is believed to be the major producer of PA involved in the regulation of most cell signaling pathways controlling cell growth and proliferation [103], AGPAT2-derived PA has been shown to link the activity of mTOR with cellular lipid contents [95,104]. Finally, PA, due to its cone shape, has the ability to distort membrane structure by causing its curvature and affecting the interaction of proteins with the membrane [105,106]. These PA properties can trigger vesicular transport and support an aggressive, invasive tumor phenotype. Indeed, PA produced on site by the AGPAT3 and AGPAT4 isoforms helps the formation of Golgi-derived vesicles [49,50,81,107]. In addition, PA was found to be enriched in exosome membranes of PC-3 prostate cancer cells [108]. Cancer cells can use these secretory vesicles and their cargo to manipulate the tumor microenvironment and establish distal pre-metastatic niches [109,110].

### 4.2. AGPAT Isoforms in Cancer

#### 4.2.1. AGPAT1

In colorectal carcinoma (CRC), expression analysis of lipid metabolism genes of patient tumor samples [82], as well as transcriptomic meta-analysis studies of CRC patient data [83,84], showed that the increased expression of AGPAT1 is associated with a high risk of relapse and shorter survival. Accordingly, AGPAT1 has been identified as a negative prognostic marker of CRC and was included in metabolic gene signatures that could help to classify CRC patients and be used for the development of precision therapies [82,83,84].

On the other hand, in a microarray analysis of transcriptomic profiles for the identification of ovarian cancer mRNA biomarkers in saliva, AGPAT1 was one of the seven validated genes, with expression levels significantly downregulated in ovarian cancer patients compared to healthy controls [85]. Although the origin and biological relevance of AGAPT1 mRNA in the saliva samples of breast cancer patients are not clarified, the downregulation of AGPAT1 mRNA was recently verified in another study for the identification of biomarkers in the saliva of ovarian cancer patients [86] and could prove helpful for establishing useful diagnostic and/or prognostic gene expression signatures in non-invasive samples from cancer patients.

#### 4.2.2. AGPAT2

Overexpression of AGAPT2 has been detected and evaluated in several tumor types and cancer cell lines. The expression of AGPAT2 mRNA and/or protein, analyzed respectively by RT-PCR and immunohistochemistry, was increased in ovarian carcinomas compared to controls [87,88,89]. Furthermore, overexpression of AGPAT2 in ovarian cancer samples was associated with aggressive histology and higher tumor grade and linked to decreased overall and disease-free survival, especially in younger patients [87,89]. Therefore, AGPAT2 could be a promising prognostic tool and possibly a target of directed therapy for ovarian cancer. In osteosarcoma, AGPAT2 expression analyzed by immunohistochemistry was increased in cancer compared to adjacent tissues [91]. In addition, AGPAT2 protein levels were higher in specimens from patients who were previously treated with cisplatin compared to patients who had not received treatment, indicating the association of AGPAT2 expression with resistance to chemotherapy [94]. Higher AGPAT2 levels were also associated with gastric cancer. A cDNA microarray analysis showed that AGPAT2 was among the genes upregulated in primary gastric cancerous tissue samples compared to tumor-adjacent tissue [90].

Epigenomic studies indicated that expression of AGPAT2 could potentially also be altered and, therefore, used as a biomarker in pediatric acute lymphoblastic leukemia (PALL). Specifically, the AGPAT2 gene was shown to be hypomethylated in datasets from PALL patients compared to normal blood donors in an integrative network analysis of differentially methylated (DMGs) and differentially expressed genes (DEGs) [92], although an earlier study did not detect any consistent differences in AGPAT2 protein levels between leukemia cell lines, cells from leukemia patients, and cells from healthy controls [111].

Moreover, AGPAT2 expression is necessary for survival and proliferation of many types of cancer cells and xenografts. Supporting the biological significance of the increased expression of AGPAT2 in ovarian cancer tissue samples, knockdown of AGPAT2 by siRNA decreased viability of ovarian cancer cells lines (SK-OV-3 and IGROV1) [88]. In addition, in osteosarcoma cells, AGPAT2 expression levels positively correlated with cell proliferation and xenograft growth [93] and an increase in cisplatin-resistant cells compared to control parental cells [94]. Furthermore, targeting of AGPAT2 by specific siRNAs or miRNAs affected cancer cell proliferation and chemoresistance. To this end, miR-24 inhibited cell proliferation in vitro and growth of xenograft tumors implanted in mice [91], while AGPAT2 silencing by specific siRNA or miR-340-5p increased apoptosis of cisplatin-resistant osteosarcoma cell lines [66]. In pancreatic cancer cells, AGPAT2 silencing resulted in the inhibition of cancer cell proliferation and anchorage-independent growth, possibly connected to the concurrent inhibition of the mTOR signaling pathway [95]. Moreover, in hepatocarcinoma (Huh7) and cervical adenocarcinoma (HeLa) cells, siRNA-mediated knockdown of AGPAT2 as well as inhibition of AGPAT2 induction by HIF-1α silencing interfere with TAG and phospholipid synthesis and prevents lipid droplet accumulation under hypoxia, resulting in a decrease of viability and increase of cancer cell sensitivity to the chemotherapeutic etoposide [27].

The profound effect of AGPAT2 expression on cancer cell proliferation suggested its possible use as a therapeutic target and led to the search and development of AGPAT2 inhibitors. A number of studies initially showed that small molecule AGAPT2 inhibitors induced apoptosis and successfully reduced cancer cell proliferation, tumor xenograft growth, and resistance to conventional chemotherapy [88,112,113,114,115,116]. However, a later study suggested that these molecules exerted their effect not specifically via inhibition of AGPAT2 but by a general inhibition of cell signaling pathways, which are activated in proliferating cells [111].

#### 4.2.3. AGPAT4

AGPAT4 was identified, together with AGPAT3, among breast cancer-expressed genes involved in endo-/exocytosis (EEC) in a reanalysis of the results from three independent genome-wide association studies [117]. These findings are in agreement with an analysis of transcriptome data from breast cancer patients that showed a correlation of low AGPAT4 expression with better prognosis [96].

In CRC patients, RNA-sequencing analysis revealed that AGPAT4 expression was increased in tumor samples compared to paracarcinoma tissues and predicted poor survival [97]. Interestingly, it was shown that silencing of AGPAT4 expression in a CRC cell line did not affect the growth or migration of CRC cells in vitro but suppressed CRC xenograft growth in mice. This appears to be, at least partly, mediated by the increased release of LPA from CRC cells upon AGPAT4 knockdown, which activates a systemic antitumor response via the shaping of the immune tumor microenvironment [97]. Specifically, the increased release of LPA from CRC cells upon AGPAT4 knockdown mediated the activation of p38/p65 pathways in macrophages, leading to macrophage stimulation, T-cell activation, and, hence, increased immune cell evasion. Consequently, the upregulation of AGPAT4 in CRC cells most likely results in the decrease of T-cell activation and suppression of antitumor immunity. Microarray analysis studies of genes regulating lipid metabolism have further shown that AGPAT4 expression is upregulated in melanoma [98] and oral squamous cell carcinoma (OSCC) [99].

In addition to AGPAT4, a non-protein-coding AGPAT4 intronic transcript, lncRNA AGPAT4-IT1 was also identified to have a potential role in cancer pathogenesis. Analysis of microarray data of neuroblastoma patients identified that expression of lncRNA AGPAT4-IT1 was associated with increased patient survival [100,101]. Likewise, integrative analysis microarray datasets from non-small cell lung cancer (NSCLC) patients showed that lncRNA AGPAT4-IT1 was downregulated in NSCLC tumors and may play an important role in NSCLC pathogenesis [102].

## 5. Conclusions

Dysregulated lipid metabolism is a hallmark of cancer and accumulating data suggest that reprogramming of the lipidome is not only a consequence of but also a driving force for cancer establishment and progression. Therefore, targeting lipid metabolism in cancer is a promising therapeutic strategy [118,119,120]. On the other hand, transcriptome analysis shows that different tumors employ specified changes of the expression of lipid metabolism genes. In the case of AGPATs, this variance is exemplified in the unique expression pattern of the family isoform enzymes in different cancer types. In projection, identification of the lipid metabolism gene signatures of different tumor types and subtypes could prove valuable for the design of targeted and effective therapeutic strategies.

## Figures and Tables

**Table 1:** Representative role of AGPAT isoforms in cancer types.

AGPAT Isoform	Cancer Type	Outcome	Ref.
AGPAT1	Colorectal carcinoma (CRC)	Increased expression of AGPAT1 is a negative prognostic marker, associated with high risk of relapse and shorter survival	[82,83,84]
	Ovarian Cancer	Downregulation of AGPAT1 mRNA levels in saliva samples from patients compared to controls	[85,86]
AGPAT2	Ovarian Cancer	Increased expression of AGPAT2 mRNA and/or protein, associated with aggressive histology, higher tumor grade, and decreased survival	[87,88,89]
	Gastric cancer	Increased AGPAT2 expression in primary gastric cancers compared to tumor-adjacent tissue samples	[90]
	Osteosarcoma	Increased AGPAT2 protein levels in cancer compared to adjacent tissues and in patients previously treated with cisplatin	[91]
	Pediatric acute lymphoblastic leukemia (PALL)	Hypomethylation of AGPAT2 gene in PALL patients compared to normal blood donors	[92]
	Ovarian cancer cells lines (SK-OV-3 and IGROV1)	Knockdown of AGPAT2 by siRNA decreased viability	[88]
	Osteosarcoma cells	AGPAT2 expression positively correlated with cell proliferation and xenograft growth and was increased in cisplatin-resistant cells compared to control parental cells. AGPAT2 silencing by specific siRNA or miR-340-5p increased apoptosis of cisplatin-resistant osteosarcoma cell lines	[66,93,94]
	Pancreatic cancer cells	AGPAT2 silencing inhibited proliferation and anchorage-independent growth	[95]
	Hepatocarcinoma (Huh7) and cervical adenocarcinoma (HeLa) cells	siRNA-mediated knockdown of AGAPT2 decreased LD accumulation under hypoxia, decreased cell viability and increased sensitivity to etoposide	[27]
AGPAT4	Breast cancer	Low AGPAT4 expression correlated with better prognosis	[96]
	Colorectal carcinoma (CRC)	Increased AGPAT4 expression in tumor samples compared to paracarcinoma tissues Increased AGPAT4 expression predicted poor survival	[97]
	Melanoma	Increased AGPAT4 expression	[98]
	Oral squamous cell carcinoma (OSCC)	Increased AGPAT4 expression	[99]
lncRNA AGPAT4-IT1	Neuroblastoma	Expression of lncRNA AGPAT4-IT1 was associated with increased patient survival	[100,101]
	Non-small cell lung cancer (NSCLC)	lncRNA AGPAT4-IT1 was downregulated in NSCLC tumors	[102]
